# Supplementary material for: Dissecting the Genetic Basis of Yield Traits and Validation of a Novel Quantitative Trait Locus for Grain Width and Weight in Rice
Source: Plants (Basel). 2024 Mar 8;13(6):770. doi: 10.3390/plants13060770 (PMC10975080; doi:10.3390/plants13060770)
Supplement: Supplementary file 1 [file plants-13-00770-s001.zip › Supplemental Figure.pptx]

## Slide 1
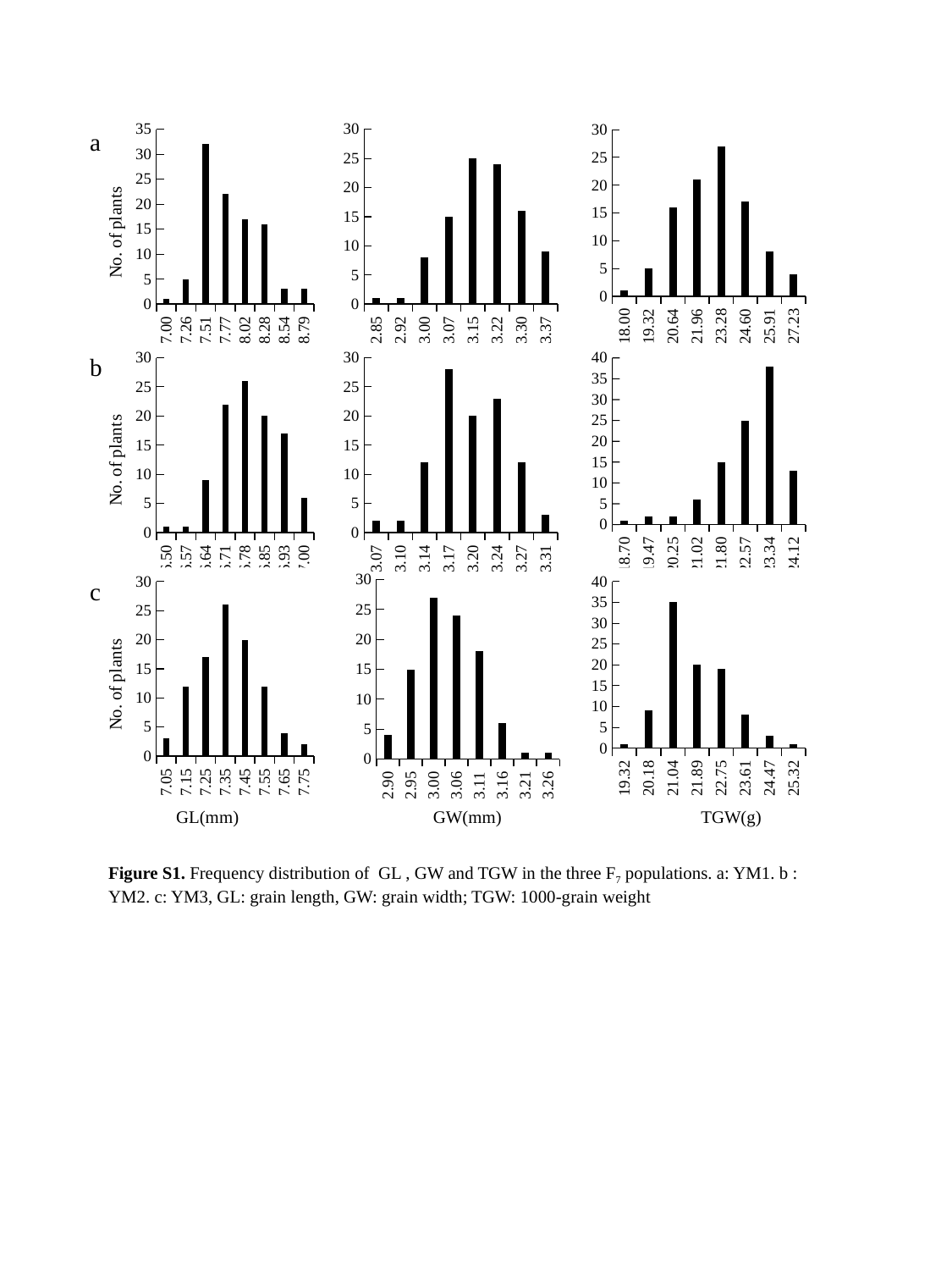

### Chart
| Category | Frequency |
|---|---|
| 7 | 1.0 |
| 7.2558571428571428 | 5.0 |
| 7.5117142857142856 | 32.0 |
| 7.7675714285714283 | 22.0 |
| 8.0234285714285711 | 17.0 |
| 8.279285714285713 | 16.0 |
| 8.5351428571428549 | 3.0 |
| 8.7909999999999968 | 3.0 |
### Chart
| Category | Frequency |
|---|---|
| 2.85 | 1.0 |
| 2.9244285714285714 | 1.0 |
| 2.9988571428571427 | 8.0 |
| 3.073285714285714 | 15.0 |
| 3.1477142857142852 | 25.0 |
| 3.2221428571428565 | 24.0 |
| 3.2965714285714278 | 16.0 |
| 3.3709999999999991 | 9.0 |
### Chart
| Category | Frequency |
|---|---|
| 18 | 1.0 |
| 19.319128571428571 | 5.0 |
| 20.638257142857142 | 16.0 |
| 21.957385714285714 | 21.0 |
| 23.276514285714285 | 27.0 |
| 24.595642857142856 | 17.0 |
| 25.914771428571427 | 8.0 |
| 27.233899999999998 | 4.0 |a
### Chart
| Category | Frequency |
|---|---|
| 6.5 | 1.0 |
| 6.5708571428571432 | 1.0 |
| 6.6417142857142863 | 9.0 |
| 6.7125714285714295 | 22.0 |
| 6.7834285714285727 | 26.0 |
| 6.8542857142857159 | 20.0 |
| 6.925142857142859 | 17.0 |
| 6.9960000000000022 | 6.0 |
### Chart
| Category | Frequency |
|---|---|
| 3.07 | 2.0 |
| 3.1037142857142856 | 2.0 |
| 3.1374285714285715 | 12.0 |
| 3.1711428571428573 | 28.0 |
| 3.2048571428571431 | 20.0 |
| 3.2385714285714289 | 23.0 |
| 3.2722857142857147 | 12.0 |
| 3.3060000000000005 | 3.0 |
### Chart
| Category | Frequency |
|---|---|
| 18.7 | 1.0 |
| 19.474128571428569 | 2.0 |
| 20.248257142857138 | 2.0 |
| 21.022385714285708 | 6.0 |
| 21.796514285714277 | 15.0 |
| 22.570642857142847 | 25.0 |
| 23.344771428571416 | 38.0 |
| 24.118899999999986 | 13.0 |b
### Chart
| Category | Frequency |
|---|---|
| 7.05 | 3.0 |
| 7.149571428571428 | 12.0 |
| 7.2491428571428562 | 17.0 |
| 7.3487142857142844 | 26.0 |
| 7.4482857142857126 | 20.0 |
| 7.5478571428571408 | 12.0 |
| 7.647428571428569 | 4.0 |
| 7.7469999999999972 | 2.0 |
### Chart
| Category | Frequency |
|---|---|
| 2.9 | 4.0 |
| 2.952 | 15.0 |
| 3.004 | 27.0 |
| 3.056 | 24.0 |
| 3.1080000000000001 | 18.0 |
| 3.16 | 6.0 |
| 3.2120000000000002 | 1.0 |
| 3.2640000000000002 | 1.0 |
### Chart
| Category | Frequency |
|---|---|
| 19.32 | 1.0 |
| 20.177742857142857 | 9.0 |
| 21.035485714285713 | 35.0 |
| 21.893228571428569 | 20.0 |
| 22.750971428571425 | 19.0 |
| 23.608714285714282 | 8.0 |
| 24.466457142857138 | 3.0 |
| 25.324199999999994 | 1.0 |c
TGW(g)
GL(mm)
GW(mm)
Figure S1. Frequency distribution of GL , GW and TGW in the three F7 populations. a: YM1. b : YM2. c: YM3, GL: grain length, GW: grain width; TGW: 1000-grain weight
