# Supplementary material for: Dissecting the Genetic Basis of Yield Traits and Validation of a Novel Quantitative Trait Locus for Grain Width and Weight in Rice
Source: Plants (Basel). 2024 Mar 8;13(6):770. doi: 10.3390/plants13060770 (PMC10975080; doi:10.3390/plants13060770)
Supplement: Supplementary file 1 [file plants-13-00770-s001.zip › Supplemental Table.docx]

**Table S1.** Sequence and physical location of the markers

| Marker | Chr. | Physical position(bp) | Forward(5'-3'） | Reverse Primer(5'-3'） |
| --- | --- | --- | --- | --- |
| YS1001 | 1 | 24240665-24241031 | ATATCCTCCTTTGATTAGCGT | GTGGGTTATTACTTTCTTCTGTT |
| YS2034 | 2 | 9224225-9224506 | CGTGGCACTCCTACAAAT | TTCTTCCAGCGAAGGTTA |
| YS2024 | 2 | 14391797-14392153 | CGGAAGAGTAAAGTGGATG | CAAGGATGTGATGGAGGA |
| YS2006 | 2 | 19241147-19240829 | ATAGTGGACCGAGTGGAGT | GCACTTATGGCTAATCACG |
| YS2027 | 2 | 20012367-20012832 | GCAGGAGTTCAGGTAGGAG | TGATCTTGCCAATCTTTGA |
| YS2010 | 2 | 23160836-23161147 | AACTCATACATTCAAGAAGCATA | CAAAAGTTGATGGACGACA |
| YS2036 | 2 | 24148523-24148836 | TTATTCCCATTTTATCCCC | TTTAATGAGACGACCCACA |
| YS2012 | 2 | 25047638-25047991 | TCCTTGGTTTACATCTTTCA | TATACTATCCCAATCCACCC |
| YS3041 | 3 | 3083497-3083867 | GACTGGAACCTCACCTCACC | GCGCACCTGTCATTATTATTCT |
| YS4002 | 4 | 6323237-6323237 | GCATCCTGTGGCTTTTACA | ATTAGGGACCTCCTGAACC |
| YS4003 | 4 | 9052774-9053083 | AAAGGATTTTCCCTAACCA | CTTGCGGATTCTATCTGTG |
| YS4005 | 4 | 17473994-17474332 | CACAATTCGGTGGTATGAGA | GATGAAAACAGAACAGGTCG |
| YS4008 | 4 | 18533264-18533264 | ATGGAGAAGAAATCCCAATA | GCAAAATAGCCTACTAATGC |
| YS4010 | 5 | 20199132-20199491 | ATGTGGGGCAGGTTTGGT | ATTCTGCATGGCGTGGTG |
| YS8006 | 8 | 2066798-2067181 | CAGGGGCTCCGAGGTCTTCT | CTTCTGGAACGCCTTTGGGT |
| YS8001 | 8 | 11387699-11388086 | CCTGGGACAAGTGTTCTACG | TTGGCAACAAGTATTTCCGT |
| YS8008 | 8 | 24112419-24112779 | TAGTCCTGCTCTTAATGGGT | TATGTCCTTTGTCCTTCTTG |
| YS8003 | 8 | 25579439-25579439 | GCCCCACCATTTGTTTTAT | CCCTCATCTATTGCCCTTG |
| YS8009 | 8 | 26110274-26110578 | GCAACCAAGAAAGGCAACCA | CTCCAGCTCAACCGAGACGT |
| YS12002 | 12 | 16297457-16297706 | AGTCGATGGCTAGATGTTTT | TATCCAATTTGTGATTCCGT |
